# Pain Phenotypes and Hematological Inflammatory Indices as Predictors of Transforaminal Epidural Steroid Injection Outcomes in Older Adults

**DOI:** 10.3390/medicina62071316

**Published:** 2026-07-08

**Authors:** Ulku Sabuncu, Gulcin Babaoglu, Sukriye Dadali, Ali Costu, Nevcihan Sahutoglu Bal, Erkan Yavuz Akcaboy

**Affiliations:** Pain Clinic, Ankara Bilkent City Hospital, Üniversiteler Mahallesi Rıfat Börekçi Caddesi No: 9, Çankaya, Ankara 06800, Turkey; gulcinpektasli@gmail.com (G.B.); sukriyedadali@gmail.com (S.D.); alicostu@gmail.com (A.C.); nevci_sahut@hotmail.com (N.S.B.); akcaboyyavuz@gmail.com (E.Y.A.)

**Keywords:** transforaminal epidural steroid injection, lumbar radicular pain, pain phenotype, neuropathic pain, nociceptive pain

## Abstract

*Background and Objectives*: Predicting treatment response following transforaminal epidural steroid injection (TFESI) in older adults with lumbar radicular pain (LRP) remains challenging. Hematological inflammatory indices have been proposed as accessible biomarkers; however, their clinical utility remains uncertain. This study aimed to evaluate the predictive value of preprocedural hematological inflammatory indices and determine whether clinical variables provide a more clinically relevant framework for predicting TFESI outcomes. *Materials and Methods*: This retrospective observational study included 190 patients aged ≥65 years who underwent TFESI for LRP. Pain intensity was assessed using the Numeric Rating Scale (NRS) at baseline and after 3 months. Treatment response was defined as a ≥50% reduction in the NRS (meaningful pain response, [MPR-50]). Pre-procedural hematological parameters and derived indices, including the neutrophil-to-lymphocyte ratio (NLR), lymphocyte-to-monocyte ratio (LMR), systemic inflammation response index (SIRI), and aggregate index of systemic inflammation (AISI), were calculated. The pain phenotype was categorized as nociceptive, neuropathic, or mixed using the Douleur Neuropathique 4 (DN4) scale. Univariate and multivariable logistic regression analyses were performed, and receiver operating characteristic (ROC) analysis was used to assess the discriminative performance. *Results*: A meaningful pain response was achieved in 61.1% of the patients. Lymphocyte and monocyte counts were higher in responders; however, effect sizes were small. Importantly, patients with neuropathic pain exhibited significantly higher monocyte counts, SIRI, and AISI, indicating an association between pain phenotype and systemic inflammatory burden; however, these markers were not associated with treatment response. Furthermore, isolated inflammatory indices demonstrated limited standalone discriminative performance in the ROC analysis. In the adjusted hematological model, NLR demonstrated a modest statistical association with MPR-50; however, this association weakened in the final integrated model. *Conclusion*: Inflammatory indices and pain phenotypes may reflect biological and clinical heterogeneity in older adults with LRP; however, their standalone predictive value for TFESI outcomes appears limited. TFESI responsiveness is likely multifactorial and may be better evaluated using an integrated clinical framework.

## 1. Introduction

Chronic low back pain (LBP), frequently accompanied by radiculopathy, is a leading cause of disability worldwide and disproportionately affects older adults. Its prevalence in individuals aged ≥60 years has been reported to range from 21% to 75%, representing a significant contributor to functional decline, healthcare utilization, and reduced quality of life [1]. With increasing life expectancy, the burden of degenerative spinal disorders is expected to increase, posing substantial clinical and socioeconomic challenges. Moreover, older adults often present with multimorbidity and age-related physiological changes, which complicate both the clinical course and treatment responses [2].

Lumbar radicular pain (LRP) is a multifactorial condition resulting from the interplay between mechanical nerve root compression and biochemical inflammatory processes [3]. In lumbar disc herniation, the displacement of the nucleus pulposus can exert direct pressure on neural structures while simultaneously triggering the release of proinflammatory mediators that sensitize nociceptive pathways. Degenerative spinal stenosis contributes to radicular symptoms through structural narrowing and chronic neural ischemia, particularly in older individuals [4].

Transforaminal epidural steroid injection (TFESI) is a widely used minimally invasive intervention for the management of LRP, enabling the targeted delivery of corticosteroids to the affected nerve root or dorsal root ganglion [3]. TFESI aims to reduce neural inflammation and alleviate pain by exerting potent anti-inflammatory effects. Although numerous studies have demonstrated its clinical efficacy, treatment outcomes remain highly variable, and identifying predictors of response continues to be a major clinical challenge, especially in the elderly population [5,6].

Aging is associated with a state of chronic low-grade inflammation, termed “inflammaging” which is driven by immunosenescence and dysregulated immune responses [7]. This altered inflammatory milieu may influence both pain perception and the effectiveness of anti-inflammatory interventions, such as corticosteroid injections.

Hematological inflammatory indices derived from routine complete blood counts, including the neutrophil-to-lymphocyte ratio (NLR), platelet-to-lymphocyte ratio (PLR), monocyte-to-lymphocyte ratio (MLR), and composite indices such as the systemic immune-inflammation index (SII), have emerged as readily available and cost-effective markers of systemic inflammation. These indices have demonstrated prognostic and predictive value in a wide range of conditions, including cardiovascular disease, malignancies, and chronic inflammatory disorders. In pain medicine, preliminary studies have suggested a potential association between these indices and pain severity or treatment outcomes; however, the evidence remains limited and largely derived from heterogeneous populations [8,9,10].

Importantly, data specifically focusing on elderly patients are limited. Given the distinct immunological profile and higher prevalence of comorbidities in this population, extrapolation from younger cohorts may not be appropriate. To date, only a limited number of studies have evaluated the predictive value of hematological inflammatory indices for TFESI outcomes exclusively in patients aged ≥65 years, and the available literature remains sparse in this regard [11].

Therefore, the primary aim of the present study was to investigate the association between preprocedural hematological inflammatory indices and clinical response following TFESI in patients aged ≥65 years. The secondary aim was to determine whether clinical variables provide a more clinically relevant framework for predicting TFESI outcomes. Identifying reliable, inexpensive, and easily accessible predictors may improve patient selection, optimize treatment strategies, and ultimately enhance clinical outcomes in this growing population of older adults.

## 2. Materials and Methods

This retrospective observational study was conducted at the Pain Clinic of Ankara Bilkent City Hospital. The study included patients aged ≥65 years who underwent TFESI for LRP between January 2022 and December 2025 at our hospital.

Patients were eligible if they had clinically and radiologically confirmed lumbar disc herniation associated with radicular pain and complete clinical, laboratory, and follow-up data. The exclusion criteria were active infection, malignancy, systemic inflammatory or autoimmune disease, hematological disorders, chronic steroid use, and missing key data variables.

The study protocol was conducted in accordance with the principles of the Declaration of Helsinki and was approved by the local ethics committee of the Scientific Research Ethics Committee of Ankara Bilkent City Hospital (Approval No: 1-26-2503; Date: 22 April 2026).

Demographic and clinical data were obtained from electronic medical records, including age, symptom duration, medication use, pain phenotype, and affected side. Pain intensity was assessed using the Numeric Rating Scale (NRS, 0–10) at baseline and 3 months after the procedure.

The structural etiology of LRP was neuroradiologically confirmed in all patients using magnetic resonance imaging (MRI). To ensure clinical characterization of this geriatric cohort, the underlying pathologies responsible for foraminal nerve root compression were systematically categorized as isolated foraminal/lateral recess stenosis, central canal stenosis with secondary foraminal extension, or acute foraminal disc herniation.

Pain phenotype classification was performed based on standardized clinical evaluations documented in electronic medical records during routine baseline assessments. Patients were categorized into nociceptive, neuropathic, or mixed pain phenotypes using a combination of clinical history, pain distribution characteristics, neurological examination findings, and DN4 (Douleur Neuropathique 4)-based neuropathic pain assessment documented in the patient files. Neuropathic pain was defined as radicular pain compatible with a dermatomal distribution accompanied by neuropathic descriptors and supportive neurological findings, including sensory alterations or positive nerve tension signs. Nociceptive pain was defined as predominantly localized axial lumbar pain without clear neuropathic or radicular features. Mixed pain was defined as the coexistence of axial nociceptive pain and neuropathic or radicular symptoms. To eliminate clinical bias, all phenotypic classifications were performed independently by two experienced pain physicians who were blinded to the patients’ pre-procedural laboratory and hematological biomarker results.

Baseline hematological parameters obtained within one week prior to the procedure included white blood cell (WBC) count, neutrophil count, lymphocyte count, monocyte count, platelet count, red cell distribution width (RDW), erythrocyte sedimentation rate (ESR), C-reactive protein (CRP), and albumin levels. In addition, several composite inflammatory indices were calculated to better reflect the systemic inflammatory status. The neutrophil-to-lymphocyte ratio (NLR) was calculated by dividing the absolute neutrophil count by the lymphocyte count and is considered a marker of systemic inflammation and stress responses. The lymphocyte-to-monocyte ratio (LMR), obtained by dividing the lymphocyte count by the monocyte count, reflects the balance between immune regulation and inflammatory activation. The systemic inflammation response index (SIRI), defined as the neutrophil count × monocyte count/lymphocyte count, represents a composite marker of innate immune activation and inflammatory burden. The aggregate index of systemic inflammation (AISI) was calculated as neutrophil × monocyte × platelet/lymphocyte, providing a more comprehensive measure of the systemic inflammatory response.

The evaluated hematological inflammatory indices were selected based on previous studies demonstrating their association with lumbar degenerative disorders, systemic inflammation, and spinal intervention outcomes. The NLR has previously been investigated as a potential biomarker for lumbar disc herniation, while aggregate inflammatory indices, such as the AISI and LMR, have been associated with disease severity and postoperative outcomes in lumbar degenerative spinal disorders. These indices are preferred because they are inexpensive, routinely available, and easily derived from standard complete blood count parameters [12,13,14].

All TFESI procedures were performed under continuous fluoroscopic guidance by experienced senior pain physicians using a standardized technique. Following standard American Society of Anesthesiologists (ASA) monitoring and established intravenous access, the patients were positioned prone with a pillow beneath the abdomen to minimize lumbar lordosis. The overlying skin was prepared with chlorhexidine and draped in a sterile manner.

**Figure 1:**
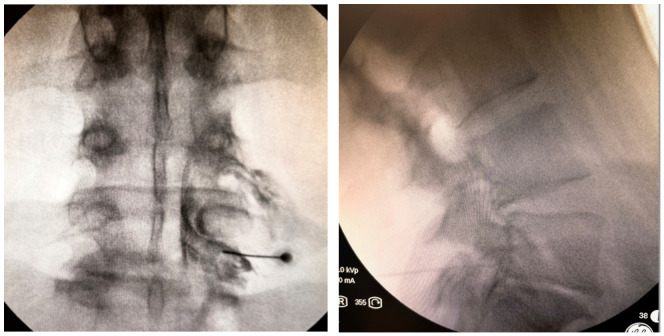
Anteroposterior and lateral fluoroscopic images showing lumbar transforaminal epidural steroid injection with appropriate needle positioning and contrast distribution around the targeted nerve root and epidural space.

Initially, the fluoroscope was adjusted in the anteroposterior projection with a cranial or caudal tilt to align the vertebral endplates. The gantry was then rotated into an ipsilateral oblique orientation (10–30°) to optimize the visualization of the target intervertebral foramen. Local infiltration was achieved using 3 mL of 2% prilocaine (Priloc; Adeka, Samsun, Turkey) solution. A 22-gauge, 3.5-inch Quincke spinal needle (Egemen International, Izmir, Turkey) was advanced unilaterally using a coaxial approach toward the subpedicular target zone at the symptomatic single level, targeting the inferior aspect of the pedicle (6 o’clock position). Correct needle placement within the epidural space was rigorously confirmed using a lateral fluoroscopic view. To ensure safe epidural delivery and prevent complications, 1–2 mL of non-ionic contrast medium (Iohexol, Biemexol; Biem İlaç, Ankara, Turkey) was injected under real-time fluoroscopy to confirm an appropriate epidural pattern and rule out vascular, subdural, or intrathecal uptake of the contrast medium. Once the target-specific perineural outline and appropriate contrast distribution were confirmed, a therapeutic mixture consisting of 1 mL normal saline, 8 mg dexamethasone sodium phosphate (Dekort; Deva Holding, Istanbul, Turkey), and 1 mL 0.25% bupivacaine hydrochloride (Buvasin; VEM İlaç, Istanbul, Turkey) was administered (Figure 1). The needle was then withdrawn, and the patients were monitored for a short period for potential immediate complications. Patients who required multilevel interventions or repeat injections during the follow-up period were excluded from the analysis to maintain cohort homogeneity.

The primary outcome was the association between hematological inflammatory indices and treatment response, defined as a ≥50% reduction in pain intensity evaluated using the NRS. A meaningful therapeutic outcome was defined as a ≥50% reduction in the Numeric Rating Scale (NRS) score at 3 months post-intervention. A ≥50% reduction in pain intensity was selected as the primary responder threshold because it is a widely accepted criterion for clinically meaningful improvement in interventional pain studies and chronic LRP literature [15,16]. Clinical outcomes were evaluated at the 3-month follow-up because this time point is commonly used in TFESI studies and represents an established intermediate-term assessment period in clinical practice. Assessing outcomes at 3 months allows evaluation beyond the immediate short-term or placebo-related effects of epidural corticosteroids while also reducing the influence of delayed, long-term degenerative progression and subsequent treatment modifications, thereby providing a relatively stable clinical assessment window in elderly patients [17,18].

Secondary outcomes included the evaluation of clinical and demographic predictors of treatment response, age, sex, symptom duration, medication use, pain phenotype, and affected side. Patients were categorized as responders and non-responders.

### Statistical Analysis

Statistical analyses were performed using IBM SPSS Statistics for Windows, Version 27.0 (IBM Corp., Armonk, NY, USA). The normality of the continuous variables was assessed using the Shapiro–Wilk test. Continuous variables are presented as mean ± standard deviation or median (interquartile range, IQR), as appropriate, whereas categorical variables are expressed as counts and percentages. For comparisons between two independent groups, the Independent Samples *t*-test was used for normally distributed variables, and the Mann–Whitney U test was applied for non-normally distributed variables. Categorical variables were compared using the chi-square test.

Changes in the NRS scores over time were analyzed using the Wilcoxon signed-rank test. Effect sizes for nonparametric comparisons were calculated using the formula r = Z/√N and interpreted as small (0.1), medium (0.3), or large (0.5). For parametric comparisons, Cohen’s d was calculated and interpreted using the standard thresholds. All statistical tests were two-tailed, and a *p*-value < 0.05 was considered statistically significant.

To identify independent predictors of a MPR-50 at 3 months, variables demonstrating a clinical or statistical trend (*p* < 0.10) in univariate analyses were entered into a multivariable binary logistic regression model using the forced entry method. Prior to model execution, the mathematical and structural stability of the covariate matrix was evaluated to rule out multicollinearity among the interrelated hematological indices (AISI, NLR, and LMR). Multicollinearity diagnostics were performed via linear dependency pathways, calculating the Variance Inflation Factors (VIF), tolerance values, and the global Condition Index. A VIF threshold of <2.5 and a Condition Index threshold of <15 were strictly applied as standard conservative criteria to confirm model stability and prevent coefficient inflation.

To comprehensively evaluate the multivariable binary logistic regression model within a supervised machine learning predictive framework, a Leave-One-Out Cross-Validation (LOOCV) protocol was utilized. Owing to the clinical sample size (*n* = 190), single partition-based train-test splits are highly susceptible to sampling instability. The LOOCV algorithm mitigates this validation bias by iteratively training the logit framework on N-1 subjects and testing its predictive accuracy blindly on the remaining single subject, repeating the cycle across the entire cohort to establish an unbiased overall classification accuracy.

A priori power analysis was conducted using G*Power version 3.1.9.7 (Heinrich-Heine-Universität Düsseldorf, Germany) to determine the required sample size for detecting differences in NLR between two independent groups. The calculation was based on the mean NLR values reported in patients with and without MPR-50 (2.34 vs. 1.79), corresponding to a moderate effect size (Cohen’s d = 0.58), as estimated by Kaya et al. [9] With a two-tailed alpha level of 0.05 and statistical power of 80%, the minimum required sample size was calculated as *n* = 96. In the present study, 190 patients were included, substantially exceeding the initial requirement. While this sample size provided sufficient power to detect the anticipated effect size, the actual observed difference in NLR within our cohort was substantially smaller than expected, which likely accounts for the lack of statistical significance despite adequate power.

A Directed Acyclic Graph (DAG) was constructed using DAGitty software, version 3.1 to guide covariate selection and minimize overadjustment bias (Supplementary Figure S1).

## 3. Results

**Figure 2:**
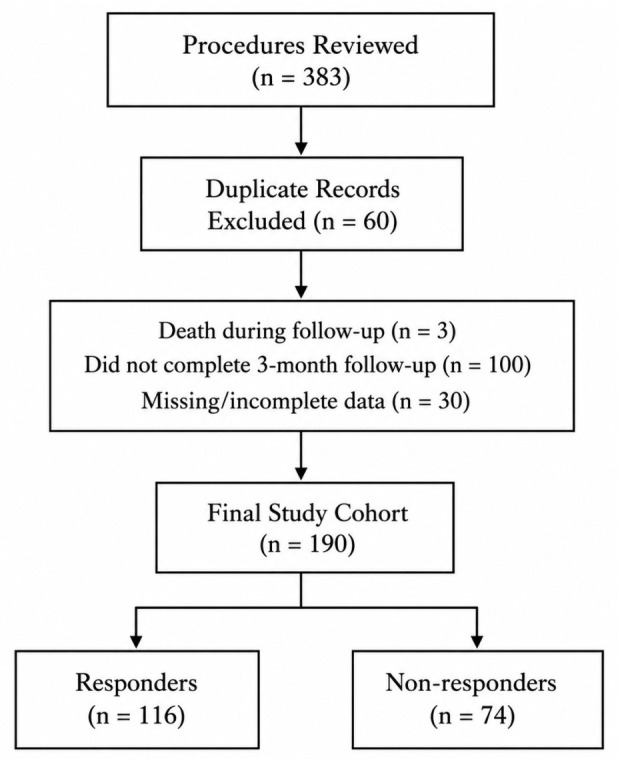
Flowchart illustrating patient screening, exclusion process, and final study cohort.

The study flow and patient selection processes are shown in Figure 2. Of the 383 procedures initially assessed, 190 patients met the inclusion criteria and were included in the final analysis after applying the predefined exclusion criteria. The cohort comprised 116 responders (61.1%) and 74 non-responders (38.9%).

The median age was 71.0 years (IQR: 67.0–77.0), with a predominance of female patients (61.1%). Mixed pain was the most common presentation (72.1%). The detailed demographic and clinical characteristics are presented in Table 1.

The median baseline NRS score was 8.0 (7.0–8.0), which significantly decreased to 4.0 (2.0–7.0) at the 3rd month (*p* < 0.001). A MPR-50, was achieved in 61.1% of the patients. The median GPE score was 6.0 (4.0–7.0) (Table 2).

Regarding the primary MRI evaluation, 114 patients (60.0%) presented with isolated foraminal/lateral recess stenosis, 52 (27.4%) with central canal stenosis accompanied by secondary foraminal extension, and 24 (12.6%) with acute foraminal disc herniations. A subanalysis revealed that the distribution of these underlying structures did not differ significantly between MPR-50 responders and non-responders (all *p* > 0.05). These findings suggest that the association observed in the multivariable NLR model (*p* = 0.034) was unlikely to be substantially influenced by differences in baseline structural pathology patterns.

**Figure 3:**
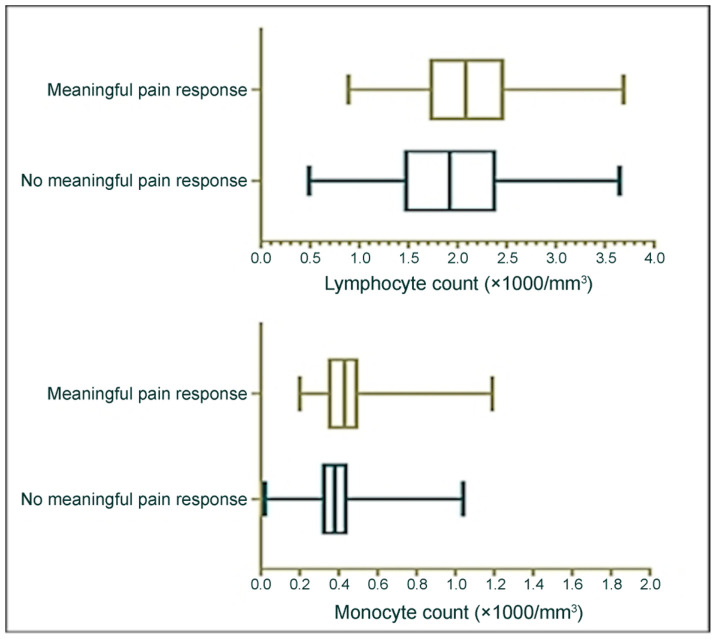
Box plots of monocyte and lymphocyte counts in responders and non-responders. The box plots show the median (central line), interquartile range (box), and minimum and maximum values (whiskers). Meaningful pain response (MPR) was defined as a ≥50% reduction in the NRS score. Cell counts are expressed as ×10^3^/m^3^.

Systemic inflammatory parameters were compared between responders and non-responders, with responders defined as those showing a decrease of ≥50% in the NRS score. Lymphocyte (*p* = 0.023) and monocyte (*p* = 0.022) counts were significantly higher in the MPR-50 group. However, the effect sizes were small (r = 0.023, 0.343, respectively). The box plot of lymphocyte and monocyte counts is presented in Figure 3. No significant differences were observed between the two groups in other inflammatory parameters (WBC, neutrophils, platelets, RDW, CRP, ESR, albumin, SIRI, AISI, NLR, and LMR) (*p* > 0.05). (Table 3). Responder ratios were found to be similar in terms of gender (Chi-square, *p* = 0.578), side of pain (Chi-square, *p* = 0.416), duration of pain (Mann–Whitney U test, *p* = 0.324, 95% CI = 256–392), and NRS-baseline (Mann–Whitney U test, *p* = 0.308, 95% CI = 241–375).

On univariate analysis screening of baseline systemic inflammatory parameters (Table 3), lymphocyte count (2.15 ± 0.60 vs. 1.95 ± 0.63 × 10^3^/mm^3^, *p* = 0.023) and monocyte count (0.43 vs. 0.38 × 10^3^/mm^3^, *p* = 0.022) were significantly higher in the MPR-50 group, with relatively small individual effect sizes (r = 0.023, 0.343, respectively). No statistically significant individual differences were observed between responders and non-responders regarding the other raw baseline hematological parameters or isolated composite indices, including NLR, LMR, SIRI, and AISI (*p* > 0.05). However, because evaluating these composite markers in isolation can hide confounding clinical paths, these primary indicators—specifically AISI, NLR, and LMR—were transitioned into a multivariable equation framework to evaluate their adjusted, simultaneous prognostic capabilities while stabilizing the structural matrix interactions.

**Figure 4:**
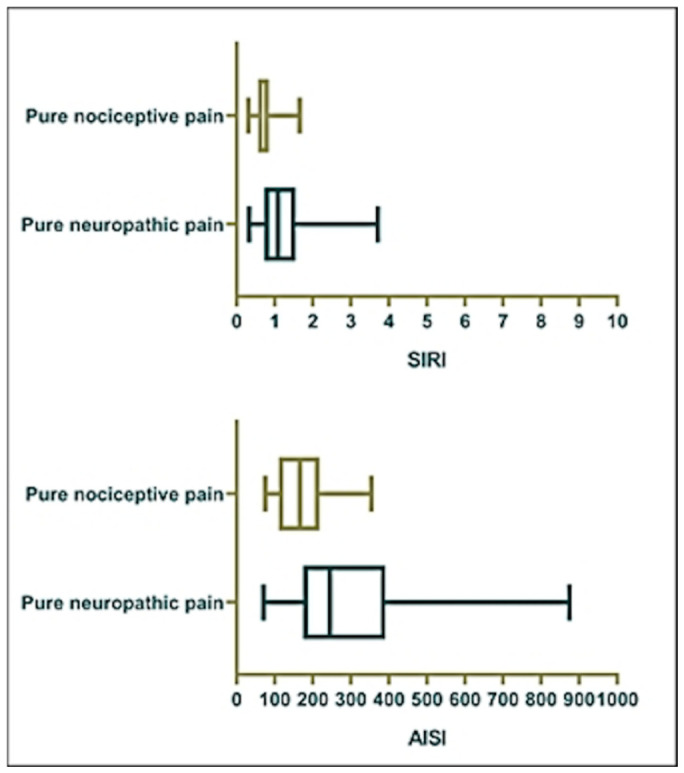
Boxplots represent the distribution of values with the median shown as the central line, boxes indicating the interquartile range (IQR), and whiskers representing the minimum and maximum values.

In the subgroup analysis, patients with purely neuropathic pain exhibited significantly higher levels of monocytes (*p* = 0.006, r = 0.38), SIRI (*p* = 0.005, r = 0.37), and AISI (*p* = 0.005, r = 0.36) than patients with purely nociceptive pain, with moderate effect sizes, suggesting a meaningful association between systemic inflammation and neuropathic pain. Box plots of the monocyte count, SIRI, and AISI are shown in Figure 4. WBC, neutrophil, lymphocyte, platelet counts, RDW, CRP, ESR, albumin, NLR, and LMR were not significantly different between patients with only nociceptive and only neuropathic pain (*p* > 0.05).

Multivariable binary logistic regression analysis demonstrated a modest statistical association between NLR and 3-month treatment response following TFESI (B = −0.696, *p* = 0.034, OR = 0.499, 95% confidence interval [CI]: 0.262–0.951). Higher baseline NLR values demonstrated a limited statistical association with a lower likelihood of achieving a MPR-50 within the adjusted hematological model. In contrast, AISI and LMR did not retain statistical significance in the multivariable model (Table 4). Collinearity diagnostics revealed low multicollinearity among the evaluated inflammatory indices. The Variance Inflation Factor (VIF) values were below 2.5 for all variables, and the maximum Condition Index remained below the predefined threshold.

**Figure 5:**
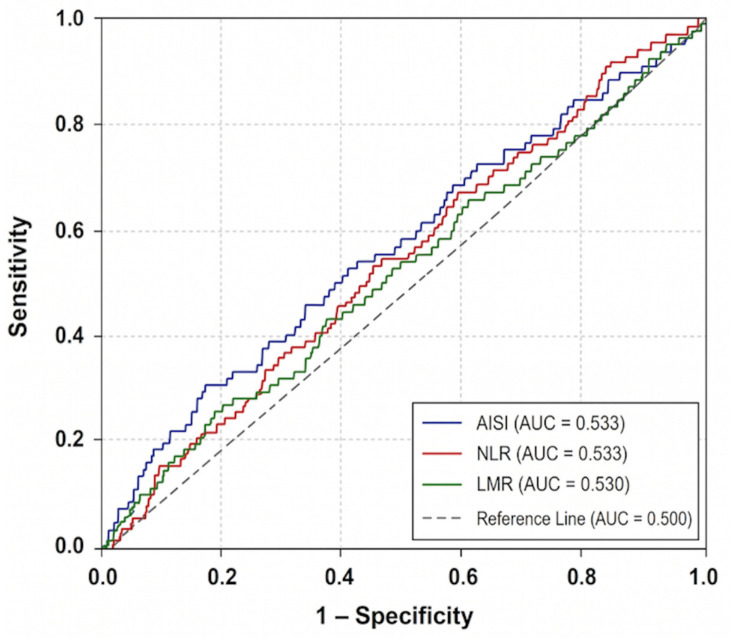
ROC curves of baseline inflammatory indices (AISI, NLR, and LMR) for predicting 3-month treatment response (MPR-50) following TFESI in older adults. The dashed diagonal line represents reference random chance (AUC = 0.500). All isolated peripheral markers track close to the reference line, demonstrating weak and statistically non-significant standalone predictive accuracy (all *p* > 0.05). This visually contrasts the limited standalone screening utility of the evaluated inflammatory markers with the limited statistical association observed for NLR within the adjusted hematological regression framework.

Receiver operating characteristic (ROC) curve analysis was performed to evaluate the standalone discriminative performance of baseline inflammatory indices for predicting MPR-50 at 3-months following TFESI (Figure 5). When analyzed individually, AISI, NLR, and LMR demonstrated limited discriminative performance, with AUC values close to 0.5 and without statistically significant standalone predictive accuracy (all *p* > 0.05). These findings suggest that isolated peripheral inflammatory markers may have limited utility as independent screening tools in routine clinical practice, despite the independent association observed for NLR in the multivariable regression model.

**Figure 6:**
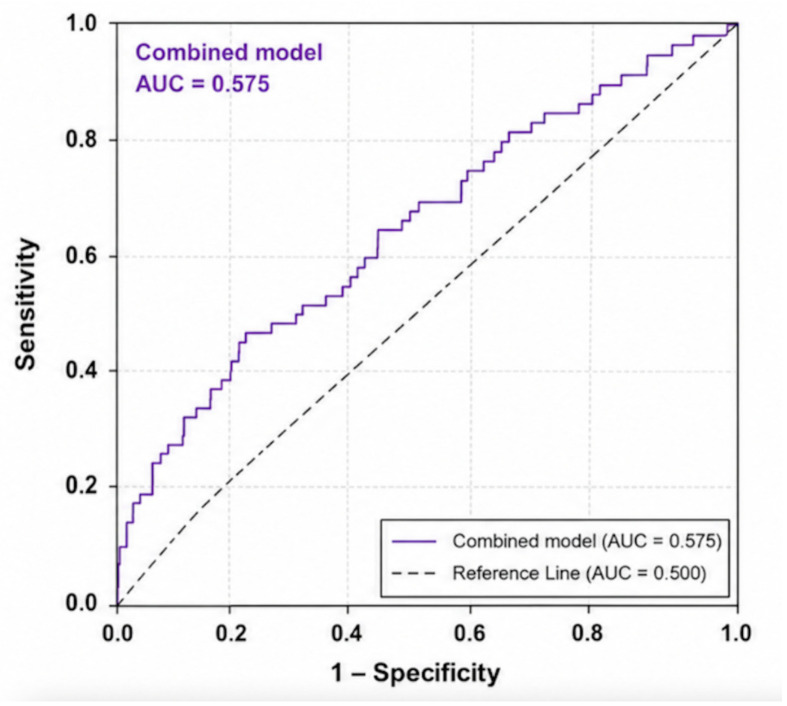
Receiver operating characteristic (ROC) curve of the final multivariable logistic regression model, including AISI, NLR, and LMR, for predicting treatment response following Transforaminal epidural steroid injection in older adults. The area under the curve (AUC = 0.575) demonstrated the limited overall discriminative performance of the adjusted inflammatory marker model.

To further evaluate the overall predictive performance of the adjusted inflammatory marker model, a ROC analysis was performed using the predicted probabilities derived from the multivariable regression equation (Figure 6). The combined model demonstrated a limited discriminative ability, with an AUC of 0.575.

To evaluate the predictive performance of the multivariable logistic regression model, a LOOCV approach was applied. Given the relatively limited cohort size (*n* = 190), LOOCV was preferred to maximize data utilization for internal validation. In this approach, the model was iteratively trained on N − 1 patients and tested on the remaining patients across all observations. The final model achieved an overall cross-validated classification accuracy of 71.1%. Nevertheless, this finding should be interpreted with caution because the discriminative performance of the model, as reflected by the AUC, remained modest.

Univariate analysis demonstrated no statistically significant differences between responders and non-responders in terms of age, pain duration, or baseline NRS scores (all *p* > 0.05). Similarly, categorical variables, including SLR, FST, MD, medication use, sex, and side of pain, were not associated with the treatment response (Table 5). However, motor deficit (MD) (χ^2^ = 3.420, *p* = 0.064) and pain type (χ^2^ = 5.736, *p* = 0.057) showed a trend toward statistical significance and were therefore included in the multivariable analysis.

The responder rates differed across the pain phenotype groups (Table 6). Patients with a mixed pain phenotype demonstrated the highest responder rate (65.7%), followed by those with neuropathic pain (54.1%), whereas those with predominantly nociceptive pain demonstrated the lowest responder rate (37.5%). The association between pain phenotype and responder status was borderline statistically significant (Pearson χ^2^ = 5.736, *p* = 0.057). However, these associations weakened after adjustment for the final integrated multivariable regression model.

In the multivariable logistic regression analysis, pain type emerged as an independent predictor of treatment response following TFESI (OR = 1.703, 95% CI: 1.067–2.718; *p* = 0.026). This finding indicates that the pain phenotype significantly influences the likelihood of achieving MPR-50. Motor deficit (MD) showed a trend toward significance (OR = 1.876, 95% CI: 0.922–3.816, *p* = 0.082), but did not reach statistical significance. Baseline pain intensity was not associated with the treatment response (*p* = 0.466).

To further evaluate the combined effects of hematological inflammatory indices and clinical variables, a final integrated multivariable logistic regression model was constructed, including AISI, NLR, LMR, motor deficit, and pain phenotype (Table 7). Within the integrated framework, neither NLR nor the other evaluated inflammatory indices demonstrated statistically significant associations with the treatment response after adjustment. The pain phenotype showed a trend toward an association with the overall treatment response (*p* = 0.102). Compared with patients presenting predominantly with nociceptive pain, those with neuropathic pain tended to demonstrate lower treatment response rates, although this association did not reach statistical significance (OR = 0.342, *p* = 0.080). Similarly, the mixed pain phenotype was not significantly associated with the treatment response after adjustment (OR = 0.556, *p* = 0.148).

## 4. Discussion

In this retrospective observational study of older adults undergoing TFESI for LRP, it was aimed to evaluate the predictive value of systemic hematological inflammatory indices for treatment response. Although several inflammatory markers demonstrated statistical associations in preliminary analyses, their overall discriminatory performance remained limited. In particular, isolated inflammatory indices demonstrated limited standalone discriminative performance, with AUC values close to the reference line. Although certain statistical associations were observed within the adjusted models, these findings weakened after the integrated analysis. Similarly, the pain phenotype showed some variability in responder distribution, with relatively higher response rates among the mixed and neuropathic pain groups than among those with predominantly nociceptive pain. Overall, these findings suggest that inflammatory markers and pain phenotypes may reflect underlying biological and clinical heterogeneity; however, their overall predictive utility appears to be limited within an integrated clinical framework. Systemic inflammatory indices, such as NLR, PLR, SIRI, and AISI, have increasingly been investigated as potential biomarkers in chronic pain disorders, including migraine, fibromyalgia, neuropathic pain, and postsurgical pain [19,20,21]. However, evidence regarding their utility in interventional pain management remains inconsistent. While some studies have reported associations between an elevated inflammatory burden and poorer treatment outcomes, others have failed to demonstrate clinically meaningful predictive value [8,9,10,11,22]. The present findings largely support the latter perspective, indicating that routine peripheral inflammatory indices alone may not provide sufficiently robust discrimination for predicting TFESI outcomes in elderly patients.

An important observation in the current study was the distinction between the statistical association and overall discriminative performance. Although certain associations were observed in the adjusted regression analyses, the overall ROC performance of the evaluated inflammatory markers remained limited, and the combined inflammatory marker model demonstrated a low discriminative ability. These findings suggest that statistically detectable associations may not necessarily translate into reliable clinical predictions. Accordingly, inflammatory biomarkers should be interpreted in conjunction with broader clinical characteristics rather than in isolation.

Inflammation is an important biological component of lumbar radicular pain and neuropathic sensitization. Persistent peripheral nociceptive signaling may promote neuroimmune activation involving macrophages, glial cells, cytokines, and chemokine-mediated sensitization pathways, thereby contributing to the maintenance of chronic pain [23]. In the present subgroup analyses, patients with purely neuropathic pain demonstrated significantly higher monocyte counts, SIRI, and AISI than those with purely nociceptive pain phenotypes, supporting the concept that systemic inflammatory activity may partially reflect differences in pain mechanisms [24,25]. Nevertheless, despite these biological distinctions, the evaluated inflammatory markers did not demonstrate stable or clinically predictive performance for the TFESI response after integrated adjustment.

Interestingly, responder distribution analyses demonstrated higher responder rates among mixed and neuropathic pain phenotypes than among those with predominantly nociceptive pain. However, these associations weakened after multivariable adjustment and did not maintain statistical significance in the final integrated regression model. This pattern suggests that the pain phenotype may contribute to treatment heterogeneity but should not be interpreted as an isolated predictor of treatment success. Furthermore, the relatively small size of the purely nociceptive subgroup and the predominance of mixed pain presentations may have influenced subgroup stability and should be considered when interpreting these results.

Several factors may account for the lack of predictive value observed in this study’s results. Pain is a complex and multifactorial phenomenon influenced by structural, neurochemical, psychological, and social determinants that cannot be fully captured by systemic inflammatory markers alone [26,27,28]. Furthermore, TFESI primarily exerts its therapeutic effect through localized anti-inflammatory mechanisms at the level of the affected nerve root, whereas hematological indices reflect the systemic inflammatory status [4,28]. This mismatch between local pathophysiology and systemic biomarkers likely limits the predictive capacity of these biomarkers. In addition, age-related immune alterations, often referred to as “inflammaging” may introduce baseline variability in inflammatory indices among older adults, further obscuring potential associations [29,30].

From a practical clinical perspective, our findings suggest that routinely available hematological inflammatory indices should not be used in isolation to determine the candidacy for TFESI in older adults with LRP. Although these biomarkers may reflect the underlying systemic inflammatory status, their limited standalone discriminative performance indicates that treatment decisions should continue to rely primarily on comprehensive clinical assessment, including pain phenotype, neurological examination, symptom characteristics, and imaging findings. Accordingly, hematological indices should be regarded as complementary rather than decisive tools for patient selection until more robust predictive models become available.

Future research should focus on developing multimodal prediction models that integrate hematological inflammatory biomarkers with MRI characteristics, neurological examination findings, pain phenotypes, and patient-reported outcome measures. Given the multifactorial nature of the treatment response following TFESI, such integrated models may provide substantially greater predictive discrimination than isolated inflammatory indices alone. Prospective multicenter studies incorporating these complementary clinical and biological domains may facilitate more accurate patient stratification and support personalized treatment selection in older adults with lumbar radicular pain.

The present study had several strengths, including a relatively large cohort of older adults, standardized fluoroscopy-guided TFESI procedures, detailed phenotypic characterization, and comprehensive evaluation of multiple inflammatory indices. In addition, the combined use of regression modeling, ROC analyses, and internal validation methods provided a more balanced assessment of both the statistical association and predictive discrimination.

This study has several limitations. First, the retrospective single-center design may introduce selection bias and residual confounding, and limit causal interpretation, despite the use of standardized institutional protocols. Of the 383 initially screened patients, 190 were included in the final analysis, while 33 patients were excluded because of unavailable laboratory data, which raised the possibility of selection bias. However, the excluded patients represented a relatively small proportion of the screened cohort, and all available eligible cases were analyzed consecutively. Second, subgroup imbalance between pain phenotypes, particularly the relatively small nociceptive subgroup, may have affected the statistical stability and limited the interpretability of subgroup comparisons. Third, although multicollinearity was formally assessed and controlled for, overlap among hematological inflammatory indices may have influenced coefficient stability within the regression models. In addition, although MRI-based structural etiologies were evaluated, no significant differences were identified between responders and non-responders in the baseline structural categories. Finally, the overall predictive performance of the evaluated inflammatory markers remained modest, highlighting the need for external validation and prospective multicenter studies incorporating multidimensional clinical, radiological, and biological predictive frameworks.

## 5. Conclusions

In conclusion, the evaluated hematological inflammatory indices and pain phenotypes demonstrated limited overall utility for predicting TFESI outcomes in older adults. Although certain statistical associations were observed in the selected analyses, these findings were weakened after integrated adjustment. Nevertheless, these parameters may still reflect the underlying biological and clinical heterogeneity relevant to the treatment response. Future studies may benefit from integrated approaches that combine clinical, radiological, and biological factors when evaluating TFESI outcomes in elderly patients.

## Figures and Tables

**Table 1:** Clinical and demographic data of patients (*n* = 190).

Age	71.0 (67.0–77.0)
Sex	
Female	116 (61.1)
Male	74 (38.9)
Side	
Right	84 (44.2)
Left	106 (55.8)
Positive SLR test	94 (49.5)
Positive FST	49 (25.8)
Motor deficit	50 (26.3)
Pain type	
Nociceptive	16 (8.4)
Neuropathic	37 (19.5)
Mixed	137 (72.1)
Pain duration	12.0 (5.0–19.5)
Medication	
NSAIDs	134 (70.5)
Gabapentinoids	22 (11.6)
Antidepressants	15 (7.9)
Opioids	11 (5.8)
None	49 (25.8)

Continuous variables are presented as median (interquartile range [IQR]), and categorical variables as number (%). SLR = straight leg raise test; FST = femoral stretch test; NSAIDs = nonsteroidal anti-inflammatory drugs.

**Table 2:** Clinical outcome scores of the patients.

NRS-Baseline	8.0 (7.0–8.0)
NRS-3rd month	4.0 (2.0–7.0)
*p*	<0.001
MPR-50	116 (61.1)
GPE score	6.0 (4.0–7.0)

Continuous variables are presented as medians (interquartile range [IQR]), and categorical variables as numbers (%). The *p* value represents the comparison between the baseline and 3-month NRS scores. NRS, Numeric Rating Scale; MPR, meaningful pain response; GPE = Global Perceived Effect; CI, confidence interval.

**Table 3:** Comparison of systemic inflammatory parameters according to meaningful pain response.

Variables	Non-Responder (*n* = 74, 38.9%)	Responder (*n* = 116, 61.1%)	*p*	95% CI
WBC (×10^3^/mm^3^)	6.83 (2.95)	7.29 (2.26)	0.170 *	0.116–0.225
Neutrophil (×10^3^/mm^3^)	4.16 (2.27)	4.41 (2.15)	0.632 *	0.562–0.702
Lymphocyte (×10^3^/mm^3^)	1.95 ± 0.63	2.15 ± 0.60	0.023 †	−0.397–0.029
Monocyte (×10^3^/mm^3^)	0.38 (0.13)	0.43 (0.15)	0.022 *	0.001–0.043
Platelet (×10^3^/mm^3^)	253.0 (107.0)	246.0 (95.0)	0.797 *	0.738–0.855
RDW	14.1 (1.4)	14.4 (1.5)	0.901 *	0.858–0.944
Sedimentation (mm/hr)	11.0 (13.5)	13.0 (14.0)	0.521 *	
CRP (mg/dL)	2.5 (6.1)	2.6 (6.7)	0.791 *	0.732–0.850
Albumin	43.0 (2.0)	42.0 (3.0)	0.989 *	0.974–1.000
SIRI	0.84 (0.73)	0.92 (0.67)	0.753 *	0.690–0.815
AISI	208.65 (223.64)	225.09 (219.66)	0.670 *	0.602–0.739
NLR	1.63 (0.90)	1.60 (0.68)	0.203 *	0.145–0.262
LMR	5.03 (2.66)	4.92 (2.35)	0.852 *	0.800–0.903

* SIRI = Systemic Inflammation Response Index; AISI = Aggregate Index of Systemic Inflammation; NLR = Neutrophil-to-Lymphocyte Ratio; LMR = Lymphocyte-to-Monocyte Ratio. Mann–Whitney U test; † Independent-samples *t*-test.

**Table 4:** Multivariable logistic regression analysis of hematological predictors of meaningful pain response following TFESI.

Variable	B	SE	*p*	OR	95% CI
NLR	−0.696	0.329	0.034	0.499	0.262–0.951
AISI	0.001	0.001	0.265	1.001	0.999–1.003
LMR	−0.079	0.108	0.464	0.924	0.748–1.142

Values are presented as regression coefficient (B), standard error (SE), *p*-value, odds ratio (OR), and 95% confidence interval (CI). The dependent variable was meaningful pain response (≥50% reduction in NRS scores) following transforaminal epidural steroid injection (TFESI). AISI = Aggregate Index of Systemic Inflammation; NLR = Neutrophil-to-Lymphocyte Ratio; LMR = Lymphocyte-to-Monocyte Ratio.

**Table 5:** Univariate analysis of clinical and demographic predictors of meaningful pain response following TFESI.

Variable	Responders (*n* = 116)	Non-Responders (*n* = 74)	Statistic	*p*
Continuous variables				
Age	—	—	U = 3734.0	0.130
Pain duration (months)	94	50	U = 2113.5	0.313
Baseline NRS	—	—	U = 3921.0	0.279
Categorical variables				
SLR	—	—	χ^2^ = 1.017	0.313
FST	—	—	χ^2^ = 1.100	0.294
MD	—	—	χ^2^ = 3.420	0.064
Pain type	—	—	χ^2^ = 5.736	0.057
Medication use	—	—	χ^2^ = 8.390	0.591
Sex	—	—	χ^2^ = 0.309	0.578
Side	—	—	χ^2^ = 0.662	0.416

Continuous variables are presented as median (interquartile range [IQR]), and categorical variables as number (%). Continuous variables were compared using the Mann–Whitney U test, and categorical variables were analyzed using Pearson’s chi-square (χ^2^) test. Statistical significance was set at *p* < 0.05. Variables with *p* < 0.10 were considered to show a trend toward significance and were included in multivariable analysis. SLR, Straight Leg Raise test; FST = Femoral Stretch Test; MD, motor deficit.

**Table 6:** Distribution of meaningful pain response according to pain phenotype.

Pain Phenotype	Non-Responder *n* (%)	Responder *n* (%)	Total
Nociceptive	10 (62.5%)	6 (37.5%)	16
Neuropathic	17 (45.9%)	20 (54.1%)	37
Mixed	47 (34.3%)	90 (65.7%)	137
Total	74 (38.9%)	116 (61.1%)	190

Responder distribution showed a borderline association across pain phenotype groups (Pearson’s chi-square χ^2^ = 5.736, *p* = 0.057).

**Table 7:** Final integrated multivariable logistic regression model for predicting meaningful pain response following TFESI.

Variable	B	SE	*p*	OR
AISI	0.001	0.001	0.450	1.001
NLR	−0.647	0.337	0.055	0.524
LMR	−0.093	0.124	0.455	0.912
Motor deficit	−0.523	0.388	0.177	0.593
Pain phenotype (overall)	—	—	0.102	—
Neuropathic pain vs. nociceptive pain	−1.073	0.612	0.080	0.342
Mixed pain vs. nociceptive pain	−0.587	0.405	0.148	0.556
Constant	2.414	1.093	0.027	11.183

Values are presented as regression coefficients (B), standard errors (SE), *p*-values, and odds ratios (OR). The dependent variable was a meaningful pain response (≥50% reduction in the NRS score) following transforaminal epidural steroid injection (TFESI). The final integrated model included hematological inflammatory indices and clinical variables that showed a trend toward significance in the univariate analysis. The pain phenotype was entered into the model using nociceptive pain as the reference category. AISI = Aggregate Index of Systemic Inflammation; NLR = Neutrophil-to-Lymphocyte Ratio; LMR = Lymphocyte-to-Monocyte Ratio.

## Data Availability

The data that support the findings of this study are available from the corresponding author upon reasonable request. The data are not publicly available due to privacy and ethical restrictions.

## Supplementary Materials

The following supporting information can be downloaded at: https://www.mdpi.com/article/10.3390/medicina62071316/s1, Figure S1: Directed acyclic graph illustrating the hypothesized causal relationships among sociodemographic factors, pain phenotype, systemic inflammation, and treatment response following transforaminal epidural steroid injection.

## Abbreviations

The following abbreviations are used in this manuscript:

AISI	Aggregate Index of Systemic Inflammation
ASA	American Society of Anesthesiologists
AUC	Area Under the Curve
CI	Confidence Interval
CRP	C-reactive Protein
DAG	Directed Acyclic Graph
DN4	Douleur Neuropathique 4
ESR	Erythrocyte Sedimentation Rate
FST	Femoral Stretch Test
GPE	Global Perceived Effect
IQR	Interquartile Range
LMR	Lymphocyte-to-Monocyte Ratio
LOOCV	Leave-One-Out Cross-Validation
LRP	Lumbar Radicular Pain
MD	Motor Deficit
MPR-50	Meaningful Pain Response
MRI	Magnetic Resonance Imaging
NLR	Neutrophil-to-Lymphocyte Ratio
NRS	Numeric Rating Scale
PLR	Platelet-to-Lymphocyte Ratio
ROC	Receiver Operating Characteristic
RDW	Red Cell Distribution Width
SE	Standard Error
SII	Systemic Immune-Inflammation Index
SIRI	Systemic Inflammation Response Index
SLR	Straight Leg Raise Test
TFESI	Transforaminal Epidural Steroid Injection
VIF	Variance Inflation Factor
WBC	White Blood Cell Count
