# Supplementary figures and images for: Pain Phenotypes and Hematological Inflammatory Indices as Predictors of Transforaminal Epidural Steroid Injection Outcomes in Older Adults
Source: Medicina (Kaunas). 2026 Jul 8;62(7):1316. doi: 10.3390/medicina62071316 (PMC13413921; doi:10.3390/medicina62071316)

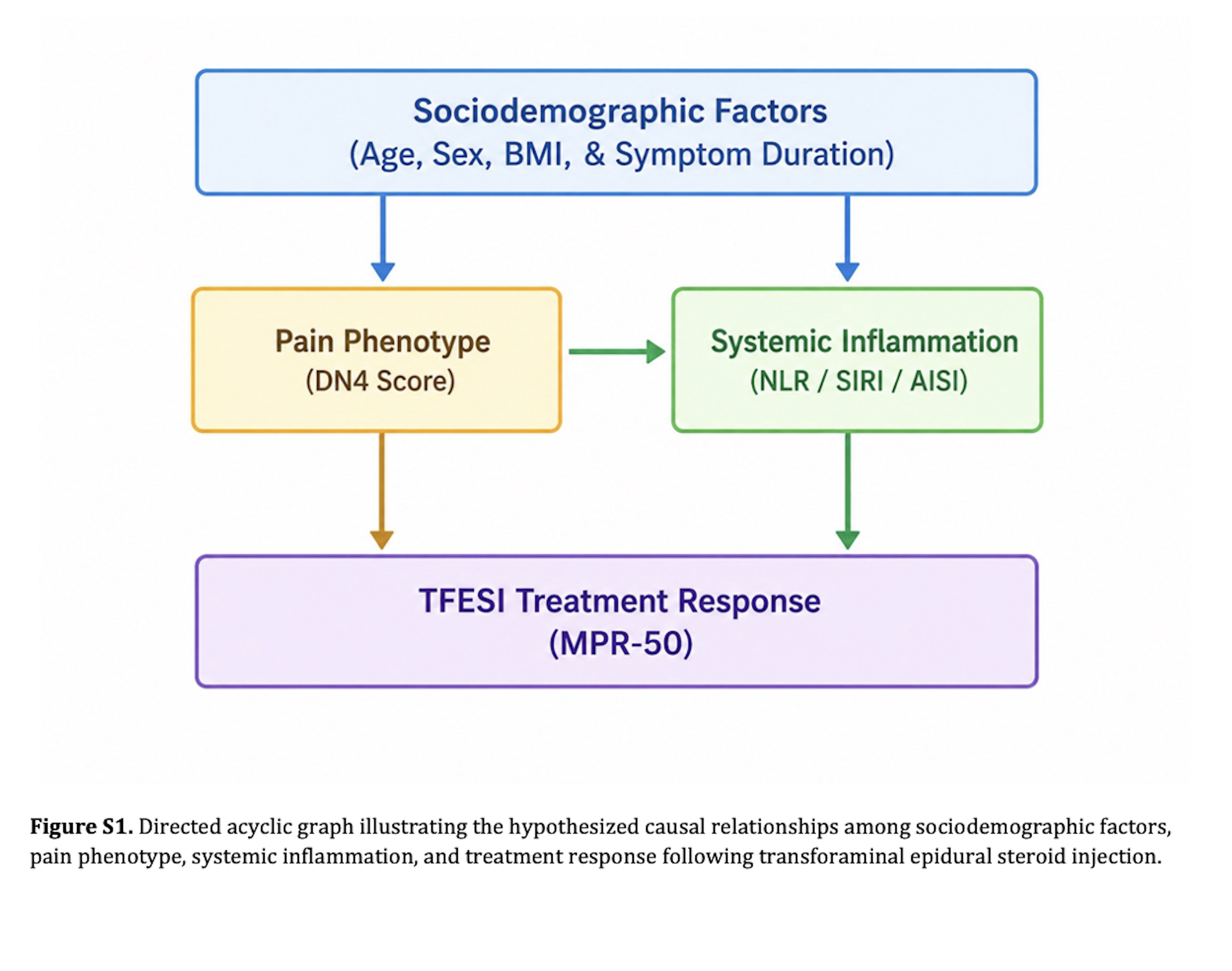

Supplement: Supplementary file 1 [file medicina-62-01316-s001.zip › medicina-4309685-supplementary.png]
